# Determination of the intraocular irradiance and potential retinal hazards at various positions in the eye during transscleral equatorial illumination for different applied pressures

**DOI:** 10.1016/j.zemedi.2022.11.005

**Published:** 2022-12-10

**Authors:** Nicole Fehler, Christian Lingenfelder, Sebastian Kupferschmid, Martin Hessling

**Affiliations:** a Institute of Medical Engineering and Mechatronics, Ulm University of Applied Sciences, Ulm, Germany; b Pharmpur GmbH, Koenigsbrunn, Germany; c Clinic of Ophthalmology, Bundeswehrkrankenhaus Ulm, Ulm, Germany

**Keywords:** Retinal hazard, Transscleral illumination, Photochemical hazard, Thermal hazard, Intraocular irradiance, Pigmentation

## Abstract

**Purpose:**

With diaphanoscopic illumination of the eye, the intensity of light entering its interior depends on the transmission properties of the eyewall. Light that passes through the eyewall can cause damage to the retina. Therefore, in this study, the intraocular irradiances are determined at different positions on the retina, directly behind the illuminated eyewall, the opposite eyewall and near the macula of ex-vivo porcine eyes. These irradiances are examined for their dependence on the pressure applied on the eyewall with the illuminating fiber and for the influence of the pigmentation of the eye.

**Methods:**

In total 221 ex-vivo porcine eyes were investigated. For transscleral illumination an illumination fiber with a diffusing adapter cap is pressed against the equatorial eyewall. The illumination fiber is pressed onto the eye and the pressure is measured in the anterior chamber. Three different pressures are applied, 23, 78 and 132 mmHg. A detection fiber with diffusing fiber tip is inserted into the eye at the desired position. The eyes were divided in groups with high and less pigmentation to investigate the influence of the pigmentation on the intraocular irradiance.

**Results:**

The intraocular irradiances E_intra_ increases for various increasing applied pressures with the illumination fiber on the eyewall and for various positions inside the eye. With this the irradiances weighted with the photochemical and thermal hazard weighting function, E_A-R_ and E_VIR-R_, also increases. Differences in E_intra_, E_A-R_ and E_VIR-R_ could be found for different pigmented eyes as these values are higher for less pigmented eyes than for strong pigmented ones.

**Conclusion:**

The hazard to the retina during diaphanoscopic illumination of the eye depends on how strong the surgeon presses the illumination fiber on the eyewall. Depending on the applied pressure and the measuring position in the eye, the specified limit for the photochemical hazard to the retina is partly exceeded. The pigmentation of the eye also plays a role. The irradiance in less pigmented eyes appears to be higher than in strongly pigmented eyes. Because of this, the surgeon should be able to adjust the intensity of the light source to the color of the patient’s eye.

## 1 Introduction

For ophthalmic surgery a bright illumination of the inside of the eye is necessary to visualize the intraocular structures. One method to illuminate the inside of the eye is diaphanoscopic illumination. Diaphanoscopy is used for a lot of applications, e.g. the detection of foreign bodies and tumors inside the eye, tumor therapy, the detection and localization of retinal tears and the results of their diathermic treatment [1], [2], [3], [4], [5], [6], [7], [8], [9], [10], [11], [12], [13], [14], [15], [16], [17], [18]. Although transscleral transmission is lower for higher pigmented eyes [19], melanomas can be diagnosed here as well [20]. An advantage of diaphanoscopy is that there are no unwanted reflexes when examining the ocular fundus, especially in eyes with small pupils and opaque media [21]. With the improvement of xenon or halogen light sources and with the integration of light guides in scleral depressors, the development of diaphanoscopic application continues to advance [22], [23], [24], [25], [26].

In addition to the low technical requirements of a diaphanoscope, patient safety is a major advantage of diaphanoscopy. No incision in the eye is necessary, in contrast to other illumination procedures like chandelier or hand-held fiber endoillumination. However, since the diaphanoscope is in direct contact with the eye in most cases, and therefore very close to the retina, potential photochemical and thermal hazards to the retina have to be considered. Thermal damage results from local heating due to visible and near-infrared light on the retina whereby the absorption of photons heats the tissue. With the application of cold light sources, in which the light appears white due to higher blue spectral components and lower red content, this hazard is reduced. But with increasing intensity of short-wavelength light the retinal photochemical hazard increases. UV and short-wavelength visible light may cause photochemical damage to the retina [27], [28], [29]. By reducing the amount of blue light in ophthalmological light sources the photochemical hazard to the retina can be decreased [28], [30], [31]. In the standard DIN EN ISO 15004-2:2007 and in Sliney et al. (2005) limit values for the photochemical and thermal hazard on the retina are given [32], [33].

With rising pressure of the illumination fiber on the eye the transmission of the eyewall increases [19], [34], [35], [36]. Therefore, more light can enter the eye and increase the intraocular irradiance. The resulting increase in intraocular irradiance at various positions in the eye and the associated photochemical and thermal hazards on the porcine retina have not yet been investigated. In this study, measurements were performed for different applied pressures of an illumination fiber with a diffusing adapter cap on the eye and irradiances were determined directly behind the eyewall, on the opposite eyewall and near the macula. The transmission property of the eyewall depends on its three layers, the sclera, the choroid and the retina. The most scattering layer is the sclera, whereas the choroid and retina are the absorbing layers. The high amount of hemoglobin and melanin in the last two layers is responsible for the low transmission for short-wavelength light due to their high absorption properties for small wavelengths [37], [38], [39]. This leads to the reddish color inside the eye during diaphanoscopic illumination. The retinal pigment epithelium (RPE) is the most absorbent tissue in the eye. However, scattering also occurs at the pigment granules [40], [41]. Since previous studies have revealed that highly pigmented eyes have higher eyewall absorption than lowly pigmented eyes [19], [37], [42], in this study the influence of pigmentation of the eye on the intraocular irradiance during diaphanoscopy was investigated.

## 2 Material and methods

### 2.1 Material

The examination was performed with ex-vivo porcine eyes. Previous studies have stated that the porcine eye is a good model for human eyes, as they have similar properties in anatomy and physiology [43], [44], [45], [46], [47], [48]. The eyes were obtained from a local slaughterhouse and stored in BSS (Balanced Salt Solution) (Alcon, Geneva, Switzerland) at 8 °C. The measurements were performed on the day of enucleation. Only eyes that revealed no signs of vitreous and eyewall opacification and no evidence of hemorrhage, as may occur during enucleation, were used. In total N = 221 porcine eyes were investigated. Since there are a lot more eyes available with brown irises compared to blue irises we used that much eyes until at least 5 eyes were examined for each investigated pressure and iris color. A detailed presentation of the number of eyes for each measurement series is given in Table 1. The eyes were divided into two groups, eyes with low pigmentation and eyes with high pigmentation. Previous studies reveal that there is a correlation between the color of the iris and the pigmentation of the fundus [49], [50], [51]. Therefore, we declared eyes with dark/brownish irises as “high pigmented” and eyes with a bluish iris as “less pigmented”. Three different measurement series were performed at three different positions inside the eye, as explained in the next section.

**Table 1:** Overview of the number of eyes for each measurement series. It is distinguished between low and high pigmented eyes.

Position	Low pigmentation			High pigmentation			Total		
	23 mmHg	78 mmHg	132 mmHg	23 mmHg	78 mmHg	132 mmHg	23 mmHg	78 mmHg	132 mmHg
1	5	5	5	14	14	15	19	19	20
2	7	8	7	28	27	28	35	35	35
3	5	6	6	14	14	13	19	20	19

### 2.2 Experimental Set-Up

**Figure 1:**
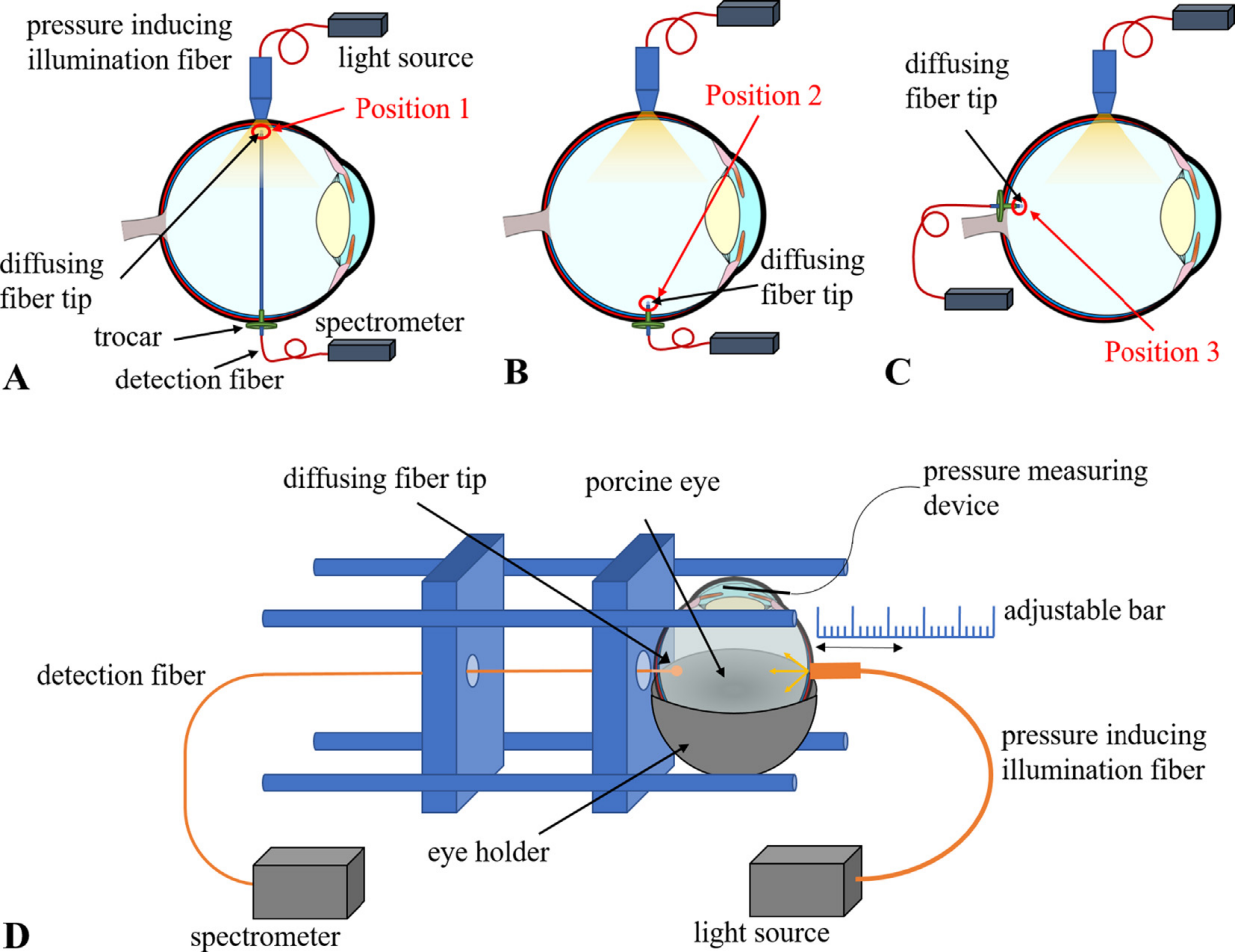
The intraocular irradiance was measured at different positions inside the eye: A) Position 1: Directly behind the eyewall opposite of the illumination fiber, B) Position 2: Behind the eyewall on the opposite side of the illumination fiber, C) Position 3: Near the macula. D) Schematic sketch of the set-up for measuring the intensity inside the eye and opposite the pressure inducing fiber tip. The illumination fiber was connected to a light source and brought in contact with the porcine eye. With the adjustable bar the fiber tip could be moved towards the porcine eye and increased the intraocular pressure, which could be measured with a tonometer with a cannula inside the anterior chamber. The detection fiber was also inserted in the eye through a cannula. With the diffusing fiber tip and homogeneous angular intensity distribution the light from all directions inside the eye could be detected and transferred to a spectrometer.

The intraocular irradiance was measured at different positions inside the eye. These positions are displayed in Fig. 1. Position 1 is located directly behind the illuminated eyewall (A), position 2 is located on the eyewall on the opposite side of the eye (B) and position 3 is in the region near the macula (C). An exemplarily schematic sketch of the measurement set-up for position 2 is illustrated in (D). The porcine eye was illuminated with an ophthalmological halogen light source Accurus Surgical System version 600 DS (Alcon Laboratories Inc., Fort Worth, TX, USA), which emits light in a spectral range between 380 and 780 nm. The light source was connected to an illumination fiber that was in contact with the sclera of the eye and therefore illuminates the sample from the outside. As diaphanoscopic illumination fiber the TotalView Endoillumination Probe, including illuminated scleral depressor (23 gauge) (D.O.R.C., Zuidland, The Netherlands) was chosen. Via an adjustable bar the fiber tip could be moved towards the sclera and brought into contact with it. If the tip was moved even further, pressure was created in the eye and the eyewall was indented. To measure the pressure rise in the anterior chamber of the eye a pressure measuring cannula was inserted through the pars plana in the anterior chamber, which was connected to a pressure transducer, Combitrans Monitoring-Set (Braun, Melsungen, Germany) via a flexible tube filled with BSS. The pressure sensor was connected to a patient monitor SMK 231 (Hellige, Freiburg, Germany). Light transmitting through the eyewall could be detected inside the eye via a detection fiber with a diffusing spherical fiber tip IP85 (Medlight, Ecublens, Switzerland). The fiber tip with a diameter of 0.85 mm was inserted into the eye through a second cannula and could detect light from all directions inside the eye. The detection fiber was connected to the spectrometer AvaSpec-HSC 1024x58TEC-EVO (Avantes, Apeldoorn, The Netherlands). The intraocular irradiances were measured for different intraocular pressures, 23, 78 and 132 mmHg. At 23 mmHg, the illumination fiber is in contact with the eye, but without applying much pressure. 132 mmHg is a very high pressure where the illumination fiber is pressed very strongly onto the eye. 78 mmHg is a value in between to study a more precise pressure dependence and not only to examine a very low and a very high pressure. These values were also selected for better comparison of previous studies of our group [19], [36]. As the spectrometer only detected the intensity inside the eye the spectrometer in combination with the detection fiber was calibrated with the calibration lamp CL2 (Bentham Instruments Limited, Reading, United Kingdom) to determine the irradiance values inside the eye. For the measurements of irradiance directly behind the eyewall, where the diaphanoscope was located, the fiber was inserted further into the eye in close proximity to the retina. For the measurement of the irradiance near the macula the part of the set-up containing the illumination fiber was rotated by 90 degrees that the illumination fiber tip was perpendicular to the detection fiber.

The calculation of the intraocular irradiance E_intra_ follows equation (1). To determine the photochemical and thermal hazard to the retina the parameters E_A-R_ and E_VIR-R_ are used according to DIN EN ISO 15004-2:2007 [32] and Sliney et al. (2005) [33]. E_A-R_ and E_VIR-R_ were calculated according to equation (2), (3), by weighting the intraocular irradiance E(λ) with the hazard weighting function A(λ) and R(λ), respectively. Both weighting functions can be taken from DIN EN ISO 15004-2:2007 [32].

(1) $$E_{\mathrm{intra}}=\sum_{380\;nm}^{1100\;nm}E\left(\lambda \right)∙\Delta \lambda$$

(2) $$E_{A-R}=\sum_{305\;nm}^{700\;nm}E\left(\lambda \right)∙A\left(\lambda \right)∙\Delta \lambda$$

(3) $$E_{\mathrm{VIR}-R}=\sum_{380\;nm}^{1400\;nm}E\left(\lambda \right)∙R\left(\lambda \right)∙\Delta \lambda$$

Applying pressure with the illumination fiber to the eye changes the transmission properties of the eyewall and thus the irradiance which enters the eye. In this study, therefore, the raise in both intraocular irradiance, ΔQ_intra_, and weighted irradiances with the photochemical and thermal hazard weighting functions, ΔQ_A-R_ and ΔQ_VIR-R_, were calculated. Two increases for E_intra_, E_A-R_ and E_VIR-R_ are calculated, the increase from 23 mmHg to 78 mmHg and from 23 mmHg to 132 mmHg. The increase was calculated according equation (4).

(4) $${\Delta Q}_{intra/A-R/VIR-R,\;\;\;78\;mmHg/132\;mmHg}=\frac{E_{intra/A-R/VIR-R,\;\;\;78\;mmHg/132\;mmHg}}{E_{intra/A-R/VIR-R,\;\;\;23\;mmHg}}$$

To distinguish between eyes with different amount of pigmentation, the ratio ΔP of the results from low pigmented eyes to high pigmented eyes was calculated, respectively for E_intra_, E_A-R_ and E_VIR-R_, for different pressures and different positions inside the eye, following equation (5).

(5) $${\Delta P}_{intra/A-R/VIR-R\;}=\frac{E_{intra/A-R/VIR-R\;}\left(less\;pigmentation\right)}{E_{intra/A-R/VIR-R}\left(high\;pigmentation\right)}$$

### 2.3 Statistical analysis

The statistical analysis was performed with SPSS Statistics (SPSS Statistics Version 25 of IBM, Armonk (USA)). To determine if there are significant differences in the results for different applied pressures, the non-parametric Kruskal-Wallis-Test was performed. To investigate whether the pigmentation of the eye has a significant effect on the results, the non-parametric Mann-Whitney-U-Test was chosen. If there was a significant effect the effect size r was calculated. The effect could be large (r ≥ 0.5), medium (0.3 ≤ r < 0.5) or small (0.1 ≤ r < 0.3). As previous analysis of the data for normal distribution showed, some data were normally distributed and some were not. Therefore, we selected a non-parametric test for all evaluations.

## 3 Results

**Figure 2:**
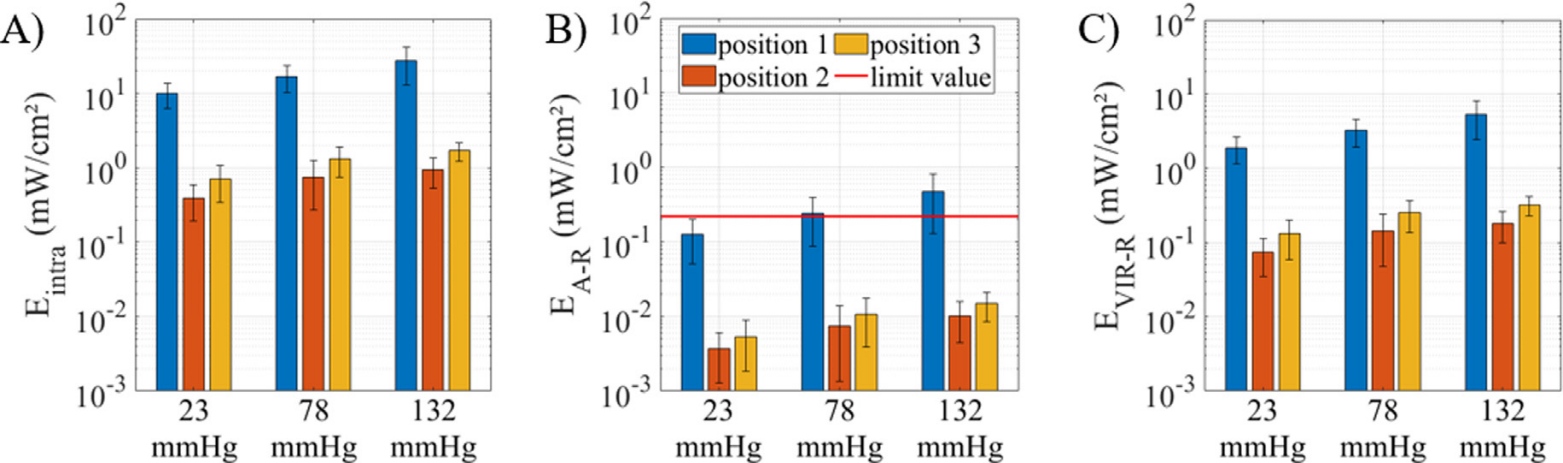
The mean values with the corresponding standard error of the mean are displayed for the intraocular irradiance, E_intra_ (A), the irradiance weighted with the photochemical hazard weighting function, E_A-R_ (B) and the irradiance weighted with the thermal hazard weighting function, E_VIR-R_ (C) at different positions inside the eye (position 1 = directly behind the eyewall opposite of the illumination fiber, position 2 = behind the eyewall on the opposite side without the illumination fiber, position 3 = near the macula) for different applied pressures on the eye with the illumination fiber tip (23, 78 and 132 mmHg).

The mean values with corresponding standard error of the mean for the intraocular irradiance E_intra_, the irradiance weighted with the photochemical hazard weighting function E_A-R_ and the irradiance weighted with the thermal hazard weighting function E_VIR-R_ are given in Fig. 2 A)-C). The blue bars indicate the results from the detection fiber at position 1, the orange bars give the results from the detection fiber at position 2 and the yellow bar show the results from the detection fiber at position 3. The red line in B) indicates the limit values of 0.22 mW/cm^2^ according to DIN EN ISO 15004-2:2007 and Sliney et al. (2005) [32], [33]. The y-axis is scaled logarithmically so that all values can be clearly seen and compared. The smallest measured values inside the eye are at position 2, where the detection fiber is located on the opposite side of the eyeball. With rising application pressure of the illumination fiber on the eye, the intraocular irradiance also rises and thus also the photochemical and thermal hazards for the retina but are all below the limit of 0.22 mW/cm^2^ and 350 mW/cm^2^ given in DIN EN ISO 15004-2:2007 [32] and in Sliney et al. (2005) [33]. The values can be taken from Table 2. At position 3 all values increase. At this position, the detection fiber is located in the area of the macula. E_intra_, E_A-R_ and E_VIR-R_ also increase with rising applied pressure on the eye by the illumination fiber. All values are below the given limits. The values at position 1 are by far the highest. The detection fiber is located directly behind the eyewall, which is dented and illuminated by the ophthalmological fiber. Here, the photochemical limit value is partly exceeded, depending on the applied pressure. However, at position 1 the limit value for the thermal hazard has not yet been reached. The increased values for E_intra_, E_A-R_ and E_VIR-R_ due to higher applied pressures on the eye are given in Table 3. By rising the pressure from 23 to 78 mmHg, the irradiance inside the eye and the associated photochemical and thermal hazards are approximately doubled. For rising the pressure from 23 to 132 mmHg the values increase even more strongly. Performing the Kruskal-Wallis-Test it could be shown that there is a significant increase in intraocular irradiance E_intra_ and in the values for the potential hazards E_A-R_ and E_VIR-R_ by increasing the applied pressure from 23 mmHg to 78 mmHg and 132 mmHg on the eye with the illumination fiber. This applies to all positions examined in the eye in this study. The results of the Kruskal-Walis-Test at each position in the eye are given in Table 5.

**Table 2:** The mean values of the intraocular irradiance, E_intra_, the irradiance weighted with the photochemical hazard weighting function, E_A-R_ and the irradiance weighted with the thermal hazard weighting function, E_VIR-R_, for different positions inside the eye and different applied pressures with the illumination fiber in comparison to the limit value from [32], [33]*.*

	Pressure [mmHg]	E_intra_ [µW/cm^2^]	E_A-R_ [µW/cm^2^]	E_VIR-R_ [µW/cm^2^]
Position 1	23	9904	125.5	1886
	78	16950	242.7	3254
	132	27490	475.3	5319
Position 2	23	394.2	3.7	73.7
	78	753.9	7.5	143.2
	132	949.1	10.1	108.6
Position 3	23	703.2	5.4	131
	78	1330	10.7	252.3
	132	1969	14.9	322.7
Limit [32], [33]		-	220	350 000

**Table 3:** Increase of the intraocular irradiance, ΔQ_intra_, the intraocular irradiance weighted with the photochemical hazard weighting function, ΔQ_A-R_, and weighted with the thermal hazard weighting function, ΔQ_VIR-R_, for a rise of intraocular pressure from 23 to 78 mmHg and from 23 to 132 mmHg for different positions of the detection fiber inside the eye. The values given are relative values, calculated according to equation (4).

Position	ΔQ_intra_		ΔQ_A-R_		ΔQ_VIR-R_	
	23 mmHg to 78 mmHg	23 mmHg to 132 mmHg	23 mmHg to 78 mmHg	23 mmHg to 132 mmHg	23 mmHg to 78 mmHg	23 mmHg to 132 mmHg
1	1.71	2.78	1.93	3.79	1.73	2.82
2	1.91	2.41	2.05	2.74	1.94	2.45
3	1.89	2.42	2.00	2.77	1.93	2.46

**Figure 3:**
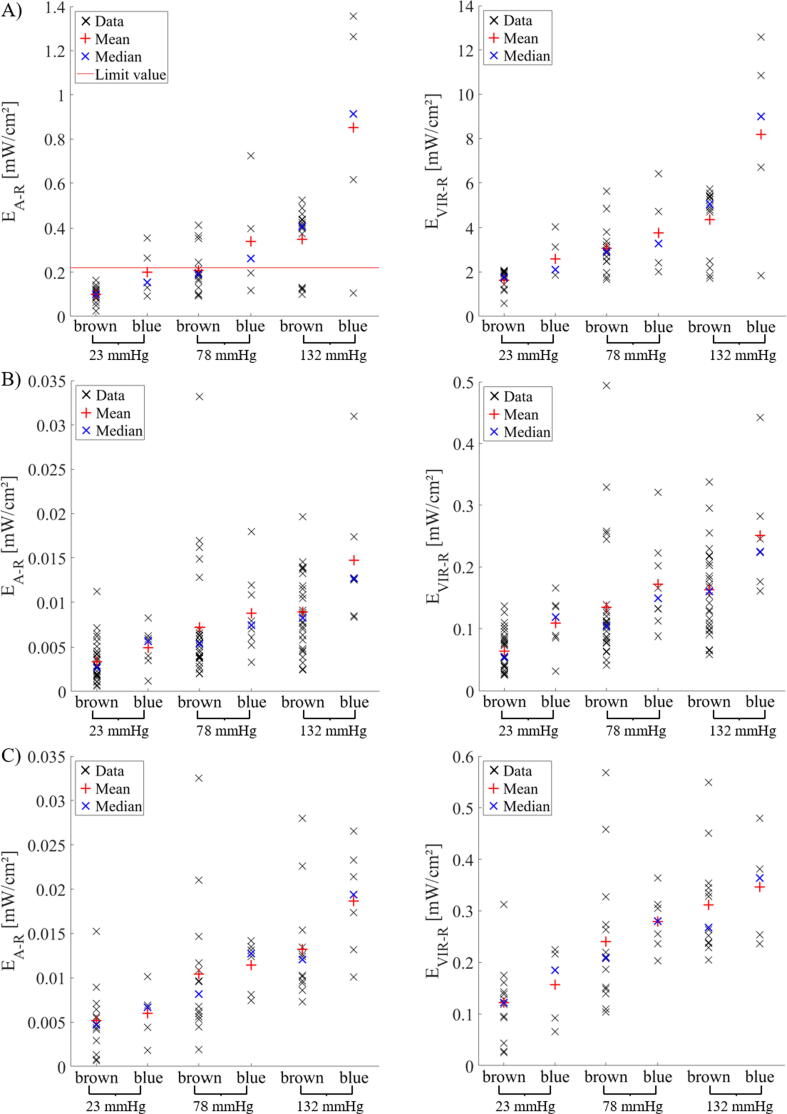
Scatter profile of E_intra_, E_A-R_ and E_VIR-R_ for eyes with less and high amount of pigmentation (blue and brown). Results are given for position 1 (A), position 2 (B) and position 3 (C). The red line in (A) indicates the limit value of the photochemical hazard.

The results in Fig. 2 and Table 3 are calculated for all eyes together (with higher and lower pigmentation). If the results for high and low pigmented eyes are considered separately, a difference can be noticed. Fig. 3 gives a scatter profile of all measured values for E_intra_, E_A-R_ and E_VIR-R_, which are shown separately for low and high amount of pigmentation (blue and brown, respectively). The results are displayed for position 1 (A), position 2 (B) and position 3 (C). The differences between the results of blue and brown eyes are calculated according to equation (5) where the ratio between the results from low pigmented eyes to high pigmented eyes are formed. In Table 4 these ratios for different applied pressures and positions are given. The photochemical hazard in the area of the macula is between 1.10 and 1.41 times higher for eyes with less pigmentation than for eyes with high pigmentation. A greater difference is observed at position 2. Here the photochemical hazard to the retina is between 1.22 and 1.66 times higher for less compared to high pigmented eyes. At position 1, directly behind the eyewall, the maximal increase in photochemical hazard for less pigmented eyes compared to high pigmented eyes is 2.43. Averaging the results of the 3 positions gives a mean increase of ΔQ_intra, mean_ = 1.41 ± 0.26, ΔQ_A-R, mean_ = 1.57 ± 0.43 and ΔQ_VIR-R, mean_ = 1.42 ± 0.27. This increase is partly significant and partly not significant depending on the position inside the eye and on the investigated parameters, E_intra_, E_A-R_ and E_VIR-R_. The results of the Mann-Whitney-U-Tests are given in Table 5. If there are significant effects the effect size was calculated and indicated with ^(+)^ for a small effect, ^(++)^ for a medium effect and ^(+++)^ for a large effect. Especially at position 3, there is no significant difference in E_intra_, E_A-R_ and E_VIR-R_ between eyes with high and small amount of pigmentation. Nevertheless, for all positions and parameters, there is a trend towards a higher retinal load in blue eyes than in brown ones.

**Table 4:** Ratio ΔP of low pigmented to high pigmented eyes for different applied pressures and positions inside the eye. The values given are relative values, calculated according to equation (5).

Position	23 mmHg			78 mmHg			132 mmHg		
	ΔP_intra_	ΔP_A-R_	ΔP_VIR-R_	ΔP_intra_	ΔP_A-R_	ΔP_VIR-R_	ΔP_intra_	ΔP_A-R_	ΔP_VIR-R_
1	1.57	2.01	1.60	1.22	1.63	1.23	1.86	2.43	1.88
2	1.68	1.48	1.69	1.28	1.22	1.28	1.53	1.66	1.54
3	1.28	1.16	1.29	1.16	1.10	1.16	1.11	1.41	1.11

**Table 5:** Overview of the results of the statistical analysis. The Kruskal-Wallis-Test is performed for determining if there are significant differences in E_intra_, E_A-R_ and E_VIR-R_ for different applied pressures (23, 78 and 132 mmHg). The Mann-Whitney-U-Test is performed to find out if there are significant differences in E_intra_, E_A-R_ and E_VIR-R_ between eyes with high or low pigmentation at different pressures. If p < 0.05 (significant effect) this is marked with yes, for p ≥ 0.05 (no significant effect) this is marked with no. A small effect is indicated with ^(+)^, a medium effect with ^(++)^ and a large effect with ^(+++)^.

**Kruskal-Wallis-Test (for pressure differences)**				**Mann-Whitney-U-Test (for pigmentation differences)**			
	E_intra_	E_A-R_	E_VIR-R_		E_intra_	E_A-R_	E_VIR-R_
mmHg	Position 1			mmHg	Position 1		
23 ↔ 78	yes ^(+++)^	yes ^(++)^	yes ^(++)^	23	yes ^(++)^	yes ^(+++)^	yes ^(++)^
23 ↔ 132	yes ^(+++)^	yes ^(+++)^	yes ^(+++)^	78	no	no	no
78 ↔ 132	no	no	no	132	yes ^(++)^	yes ^(++)^	yes ^(++)^
	Position 2				Position 2		
23 ↔ 78	yes ^(++)^	yes ^(++)^	yes ^(++)^	23	yes ^(++)^	no	yes ^(++)^
23 ↔ 132	yes ^(+++)^	yes ^(+++)^	yes ^(+++)^	78	yes ^(++)^	no	yes ^(++)^
78 ↔ 132	no	yes ^(+)^	yes ^(+++)^	132	yes ^(++)^	yes ^(++)^	no
	Position 3				Position 3		
23 ↔ 78	yes ^(+++)^	yes ^(++)^	yes ^(+++)^	23	no	no	no
23 ↔ 132	yes ^(+++)^	yes ^(+++)^	yes ^(+++)^	78	no	no	no
78 ↔ 132	no	no	no	132	no	no	no

## 4 Discussion

Our results reveal that the intraocular irradiance, E_intra_, increases with rising pressure applied to the eye with the ophthalmic illumination fiber. The increase in irradiance occurs at all 3 investigated positions in the eye (directly behind the eyewall, on the opposite side of the inner eye and in the macular region). With increasing E_intra_ the photochemical and thermal hazard to the retina also increase. In contrast to the thermal hazard, where all values at all positions and pressures are below the limit of 350 mW/cm^2^, the photochemical hazard exceeds the limit value of 0.22 mW/cm^2^ depending on the location in the eye. The photochemical hazard to the retina is highest on the directly adjacent side of the eyewall on which the illumination fiber is placed, decreases in the area of the macula and is the lowest on the opposite eyewall.

In this study, the ophthalmic illumination fiber was placed on the eyewall at the equatorial region. When the position of the fiber is changed, the amount of light entering the inner part of the eye also changes. The thickness of the sclera and the melanin concentration in the eyewall depends on the location in the eye. Therefore, the transmission properties will vary depending on the location of the light guide on the eyewall. There is also a large scattering of the thickness and melanin content in the eyewall between different eyes. This is the reason why the data in Fig. 3 show a large scatter. Despite this strong scattering it can be observed that the intraocular irradiance is higher in eyes with blue irises than in eyes with brown irises, because there is more blue absorbing melanin in brown eyes than in blue eyes. A statistical analysis of the data reveals different results for different positions and investigated parameters. In some cases, eye color has a significant influence on the level of intraocular irradiance and on the associated potential retinal hazards. However, this is not always the case. Near the macula, for example, E_intra_, E_A-R_ and E_VIR-R_ do not differ significantly at different degrees of pigmentation. However, Table 4 shows that less pigmented eyes trend to have more light measured inside the eye than in more pigmented eyes. This is an important “take home message” for the ophthalmologist. He should keep in mind that with blue eyes more light reaches the inside of the eye than with dark eyes. Therefore, the intensity of the illumination should be lowered somewhat for blue/light eyes, since the same intensity can cause more damage in blue eyes than in brown eyes.

Fig. 2 illustrates how risks vary in specific areas of the retina. Table 3 and Fig. 2 reveal the dangers that can result from excessive pressure on the eye with the illumination fiber. Since humans have a lower melanin content in the eye than pigs [49], [52], the values measured in this study, E_intra_, E_A-R_ and E_VIR-R_ , are probably even higher for humans. Less light is absorbed by the eyewall and thus more light can reach the inside of the human eye. The same effect should be caused due to the difference scleral thickness. Since porcine sclera is about twice as thick as human sclera [43], more light is scattered and absorbed. Therefore, the intraocular irradiances and the retinal hazards determined in this study are lower than they actually would be in humans. It can be assumed that the same measurements on human eyes would lead to higher results for E_intra_, E_A-R_ and E_VIR-R_ due to the smaller scleral thickness. Due to many anatomical and physiological similarities between porcine and human eyes we assume that the results of this study can be transferred to human ones, but real measurements on humane eyes should be performed to verify the results of this study.

The values for E_intra_, E_A-R_ and E_VIR-R_ have been determined for one combination of ophthalmic light source and illumination fiber with diffusing cap for diaphanoscopic application. If another light source would be applied, for example with higher blue content, the potential photochemical hazard to the retina could increase. In general, the retinal risk strongly depends on the emission spectrum of the lamp and on the transmission properties of the illumination fiber, as well as of the size of the diaphanoscope tip and on its radiation pattern. Additionally, this study reveals that the risk also depends on the amount of pigmentation in the eyewall and on the applied pressure with the illumination fiber on the eye.

## 5 Conclusion

When using transscleral illumination of the eye to detect tumors or retinal tears, it is important to know the intraocular irradiance and the associated damage to the retina. Depending on the investigated position in the eye and the applied pressure with the illumination fiber on the eye, the indicated limit value for the photochemical hazard to the retina is partly exceeded. The highest irradiances were measured on the adjacent side of the eyewall, which is illuminated and indented with the illumination fiber. The smallest values were measured at the opposite eye wall, and the values in the macular region were in between. These three studied positions could be the basis for a map showing the surgeon the spots to pay attention to where the lighting could be hazardous to the retina. The pressure-dependent measurements reveal that the retinal risk increases with higher pressure, independent of the measuring position inside the eye. Another influence on the risk is the pigmentation of the eye. The irradiance in less pigmented eyes appears to be higher than in strong pigmented eyes. This fact should be known to the surgeon. He should be able to adjust the intensity of the light source to the color of the patient’s eye. However, our study is limited to porcine eyes only. To give a relevant statement for the operator, measurements on in-vivo human eyes are needed.

## Funding

This work was financially supported by the German Federal Ministry of Economics and Technology within the ZIM joint project “Safe Light” (grant number ZF4137902AK9).

## Declaration of Competing Interest

The authors declare that they have no known competing financial interests or personal relationships that could have appeared to influence the work reported in this paper.
